# 5-Chloro-8-hydroxy­quinolinium nitrate

**DOI:** 10.1107/S160053680901993X

**Published:** 2009-05-29

**Authors:** Seik Weng Ng

**Affiliations:** a Department of Chemistry, University of Malaya, 50603 Kuala Lumpur, Malaysia

## Abstract

The 5-chloro-8-hydroxy­quinolinium cation in the the title ion pair, C_9_H_7_ClNO^+^·NO_3_^−^, is approximately coplanar with the nitrate anion [dihedral angle = 16.1 (1)°]. Two ion pairs are hydrogen bonded (2 × O—H⋯O and 2 × N—H⋯O) about a center of inversion, generating an *R*_4_^4^(14) ring.

## Related literature

The 8-hydroxy­quinolinium cation has been isolated as a number of salts; for the 8-hydroxy­quinolinium chloride hydrate, see: Skakle *et al.* (2006 ▶). For the crystal structure of 5-chloro-8-hydroxy­quinoline, see: Banerjee & Saha (1986 ▶).
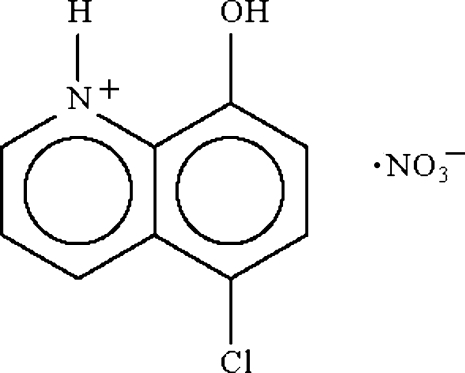


## Experimental

### 

#### Crystal data

C_9_H_7_ClNO^+^·NO_3_^−^
*M* *_r_* = 242.62
Monoclinic, 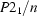

*a* = 7.4379 (3) Å
*b* = 11.5518 (6) Å
*c* = 11.2288 (5) Å
β = 95.831 (3)°
*V* = 959.80 (8) Å^3^
*Z* = 4
Mo *K*α radiation
μ = 0.40 mm^−1^
*T* = 123 K
0.20 × 0.05 × 0.05 mm

#### Data collection

Bruker SMART APEX diffractometer
Absorption correction: multi-scan (*SADABS*; Sheldrick, 1996 ▶) *T*_min_ = 0.925, *T*_max_ = 0.980
6472 measured reflections
2196 independent reflections
1574 reflections with *I* > 2˘*I*)
*R*_int_ = 0.049

#### Refinement

*R*[*F* ^2^ > 2σ(*F* ^2^)] = 0.062
*wR*(*F* ^2^) = 0.192
*S* = 1.07
2196 reflections
153 parameters
2 restraints
H atoms treated by a mixture of independent and constrained refinement
Δρ_max_ = 1.20 e Å^−3^
Δρ_min_ = −0.34 e Å^−3^

### 

Data collection: *APEX2* (Bruker, 2008 ▶); cell refinement: *SAINT* (Bruker, 2008 ▶); data reduction: *SAINT*; program(s) used to solve structure: *SHELXS97* (Sheldrick, 2008 ▶); program(s) used to refine structure: *SHELXL97* (Sheldrick, 2008 ▶); molecular graphics: *X-SEED* (Barbour, 2001 ▶); software used to prepare material for publication: *publCIF* (Westrip, 2009 ▶).

## Supplementary Material

Crystal structure: contains datablocks global, I. DOI: 10.1107/S160053680901993X/tk2465sup1.cif

Structure factors: contains datablocks I. DOI: 10.1107/S160053680901993X/tk2465Isup2.hkl

Additional supplementary materials:  crystallographic information; 3D view; checkCIF report

## Figures and Tables

**Table 1:** Hydrogen-bond geometry (Å, °)

*D*—H⋯*A*	*D*—H	H⋯*A*	*D*⋯*A*	*D*—H⋯*A*
O1—H1o⋯O2^i^	0.84 (1)	1.87 (1)	2.695 (3)	169 (4)
N1—H1n⋯O2	0.88 (1)	1.95 (1)	2.816 (3)	167 (4)

Symmetry code: (i) 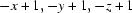.

**Fig. 1.**
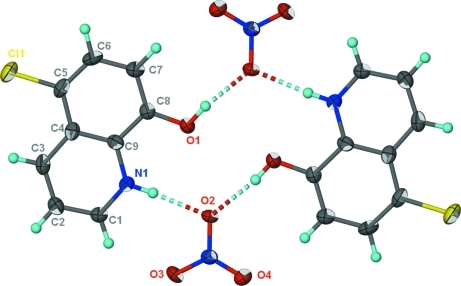
Thermal ellipsoid plot (Barbour, 2001) of a pair of hydrogen-bonded [C9H7ClNO][NO3] ion pairs drawn at the 70% probability level. Hydrogen atoms are drawn spheres of arbitrary radius and dashed lines denote hydrogen bonds.

## supplementary crystallographic information

### Experimental

Zinc acetate (0.19 g, 1 mmol) and 5-chloro-8-hydroxyquinoline (0.36 g, 2 mmol) were loaded into a convection tube; the tube was filled with dry methanol and kept at 333 K. Yellow crystals were collected from the side-arm after several days.

### Refinement

Carbon-bound H-atoms were placed in calculated positions (C–H 0.95 Å) and were included in the refinement in the riding model approximation with *U*(H) fixed at 1.2*U*(C). The ammonium and hydroxy H-atoms were located in a difference Fourier map, and were refined with distance restraints of N–H = 0.88±0.01 Å and O–H = 0.84±0.01 Å; their isotropic temperature factors were refined.

The final difference Fourier map had a large peak at about 1 Å from the Cl1 atom.

### Crystal data

**Table:**

C_9_H_7_ClNO^+^·NO_3_^−^	*F*(000) = 496
*M**_r_* = 242.62	*D*_x_ = 1.679 Mg m^−^^3^
Monoclinic, *P*2_1_/*n*	Mo *K*α radiation, λ = 0.71073 Å
Hall symbol: -P 2yn	Cell parameters from 1079 reflections
*a* = 7.4379 (3) Å	θ = 2.5–26.4°
*b* = 11.5518 (6) Å	µ = 0.40 mm^−^^1^
*c* = 11.2288 (5) Å	*T* = 123 K
β = 95.831 (3)°	Prism, yellow
*V* = 959.80 (8) Å^3^	0.20 × 0.05 × 0.05 mm
*Z* = 4	

### Data collection

**Table:**

Bruker SMART APEX diffractometer	2196 independent reflections
Radiation source: fine-focus sealed tube	1574 reflections with *I* > 2˘*I*)
graphite	*R*_int_ = 0.049
ω scans	θ_max_ = 27.5°, θ_min_ = 2.5°
Absorption correction: multi-scan (*SADABS*; Sheldrick, 1996)	*h* = −9→9
*T*_min_ = 0.925, *T*_max_ = 0.980	*k* = −15→12
6472 measured reflections	*l* = −14→14

### Refinement

**Table:**

Refinement on *F*^2^	Primary atom site location: structure-invariant direct methods
Least-squares matrix: full	Secondary atom site location: difference Fourier map
*R*[*F*^2^ > 2σ(*F*^2^)] = 0.062	Hydrogen site location: inferred from neighbouring sites
*wR*(*F*^2^) = 0.192	H atoms treated by a mixture of independent and constrained refinement
*S* = 1.07	*w* = 1/[σ^2^(*F*_o_^2^) + (0.1164*P*)^2^ + 0.1797*P*] where *P* = (*F*_o_^2^ + 2*F*_c_^2^)/3
2196 reflections	(Δ/σ)_max_ = 0.001
153 parameters	Δρ_max_ = 1.20 e Å^−^^3^
2 restraints	Δρ_min_ = −0.34 e Å^−^^3^

### Fractional atomic coordinates and isotropic or equivalent isotropic displacement parameters (Å^2^)

**Table:**

	*x*	*y*	*z*	*U*_iso_*/*U*_eq_	
Cl1	−0.30656 (11)	0.30338 (8)	0.76985 (8)	0.0321 (3)	
O1	0.3429 (3)	0.4706 (2)	0.5632 (2)	0.0279 (6)	
H1O	0.350 (6)	0.5423 (11)	0.553 (4)	0.042 (12)*	
O2	0.5861 (3)	0.30211 (18)	0.4592 (2)	0.0261 (6)	
O3	0.6434 (3)	0.12125 (19)	0.4947 (2)	0.0325 (6)	
O4	0.8190 (3)	0.2201 (2)	0.3904 (2)	0.0286 (6)	
N1	0.2829 (4)	0.2424 (2)	0.5774 (2)	0.0203 (6)	
H1N	0.385 (3)	0.265 (4)	0.552 (3)	0.037 (11)*	
N2	0.6849 (4)	0.2125 (2)	0.4472 (2)	0.0220 (6)	
C1	0.2635 (4)	0.1291 (3)	0.5806 (3)	0.0250 (7)	
H1A	0.3516	0.0806	0.5504	0.030*	
C2	0.1155 (5)	0.0794 (3)	0.6278 (3)	0.0277 (7)	
H2	0.1012	−0.0023	0.6295	0.033*	
C3	−0.0094 (4)	0.1513 (3)	0.6720 (3)	0.0265 (7)	
H3	−0.1093	0.1185	0.7063	0.032*	
C4	0.0084 (4)	0.2727 (3)	0.6672 (3)	0.0213 (7)	
C5	−0.1144 (4)	0.3518 (3)	0.7089 (3)	0.0254 (7)	
C6	−0.0849 (5)	0.4687 (3)	0.7003 (3)	0.0325 (8)	
H6	−0.1694	0.5213	0.7282	0.039*	
C7	0.0666 (5)	0.5118 (3)	0.6513 (3)	0.0297 (8)	
H7	0.0832	0.5931	0.6460	0.036*	
C8	0.1920 (4)	0.4385 (3)	0.6108 (3)	0.0231 (7)	
C9	0.1613 (4)	0.3178 (3)	0.6184 (3)	0.0204 (7)	

### Atomic displacement parameters (Å^2^)

**Table:**

	*U*^11^	*U*^22^	*U*^33^	*U*^12^	*U*^13^	*U*^23^
Cl1	0.0219 (5)	0.0405 (6)	0.0359 (5)	−0.0012 (3)	0.0128 (3)	−0.0003 (4)
O1	0.0275 (13)	0.0173 (12)	0.0413 (14)	0.0001 (9)	0.0147 (10)	0.0025 (10)
O2	0.0276 (13)	0.0138 (11)	0.0388 (13)	0.0032 (9)	0.0124 (10)	−0.0010 (9)
O3	0.0337 (14)	0.0145 (11)	0.0513 (16)	−0.0003 (9)	0.0135 (12)	0.0044 (10)
O4	0.0270 (13)	0.0265 (13)	0.0342 (13)	0.0027 (9)	0.0121 (10)	−0.0009 (10)
N1	0.0173 (14)	0.0205 (13)	0.0235 (14)	0.0010 (10)	0.0046 (11)	0.0003 (10)
N2	0.0214 (14)	0.0175 (13)	0.0274 (14)	0.0005 (10)	0.0038 (11)	−0.0025 (10)
C1	0.0241 (17)	0.0199 (16)	0.0320 (18)	0.0020 (12)	0.0073 (13)	−0.0003 (13)
C2	0.0278 (17)	0.0205 (16)	0.0356 (18)	−0.0003 (13)	0.0066 (14)	0.0058 (14)
C3	0.0227 (17)	0.0272 (17)	0.0300 (17)	−0.0079 (13)	0.0043 (13)	0.0027 (13)
C4	0.0208 (16)	0.0253 (16)	0.0180 (15)	−0.0034 (12)	0.0034 (12)	0.0000 (12)
C5	0.0182 (16)	0.0337 (19)	0.0252 (16)	−0.0003 (13)	0.0074 (12)	0.0025 (13)
C6	0.0271 (18)	0.0279 (18)	0.045 (2)	0.0074 (14)	0.0137 (15)	−0.0051 (15)
C7	0.0291 (19)	0.0210 (17)	0.0405 (19)	0.0021 (13)	0.0111 (15)	−0.0024 (14)
C8	0.0240 (16)	0.0201 (16)	0.0258 (16)	−0.0012 (12)	0.0058 (12)	0.0006 (12)
C9	0.0239 (17)	0.0171 (15)	0.0203 (15)	0.0021 (12)	0.0021 (12)	0.0009 (11)

### Geometric parameters (Å, °)

**Table:**

Cl1—C5	1.739 (3)	C2—H2	0.9500
O1—C8	1.343 (4)	C3—C4	1.410 (5)
O1—H1O	0.84 (1)	C3—H3	0.9500
O2—N2	1.285 (3)	C4—C5	1.405 (4)
O3—N2	1.234 (3)	C4—C9	1.411 (4)
O4—N2	1.240 (4)	C5—C6	1.374 (5)
N1—C1	1.318 (4)	C6—C7	1.395 (5)
N1—C9	1.368 (4)	C6—H6	0.9500
N1—H1N	0.88 (1)	C7—C8	1.371 (4)
C1—C2	1.393 (5)	C7—H7	0.9500
C1—H1A	0.9500	C8—C9	1.417 (4)
C2—C3	1.375 (5)		
			
C8—O1—H1O	113 (3)	C5—C4—C3	124.6 (3)
C1—N1—C9	123.0 (3)	C9—C4—C3	117.7 (3)
C1—N1—H1N	114 (3)	C6—C5—C4	120.1 (3)
C9—N1—H1N	123 (3)	C6—C5—Cl1	119.2 (3)
O3—N2—O4	122.1 (3)	C4—C5—Cl1	120.7 (3)
O3—N2—O2	118.2 (3)	C5—C6—C7	121.3 (3)
O4—N2—O2	119.7 (3)	C5—C6—H6	119.3
N1—C1—C2	120.8 (3)	C7—C6—H6	119.3
N1—C1—H1A	119.6	C8—C7—C6	120.9 (3)
C2—C1—H1A	119.6	C8—C7—H7	119.5
C3—C2—C1	118.5 (3)	C6—C7—H7	119.5
C3—C2—H2	120.7	O1—C8—C7	125.8 (3)
C1—C2—H2	120.7	O1—C8—C9	116.3 (3)
C2—C3—C4	121.2 (3)	C7—C8—C9	118.0 (3)
C2—C3—H3	119.4	N1—C9—C4	118.8 (3)
C4—C3—H3	119.4	N1—C9—C8	119.3 (3)
C5—C4—C9	117.8 (3)	C4—C9—C8	121.9 (3)
			
C9—N1—C1—C2	−0.5 (5)	C6—C7—C8—O1	−179.4 (3)
N1—C1—C2—C3	−0.5 (5)	C6—C7—C8—C9	1.0 (5)
C1—C2—C3—C4	1.6 (5)	C1—N1—C9—C4	0.5 (4)
C2—C3—C4—C5	179.2 (3)	C1—N1—C9—C8	−179.6 (3)
C2—C3—C4—C9	−1.7 (5)	C5—C4—C9—N1	179.8 (3)
C9—C4—C5—C6	0.8 (5)	C3—C4—C9—N1	0.6 (4)
C3—C4—C5—C6	179.9 (3)	C5—C4—C9—C8	−0.2 (4)
C9—C4—C5—Cl1	179.8 (2)	C3—C4—C9—C8	−179.4 (3)
C3—C4—C5—Cl1	−1.1 (5)	O1—C8—C9—N1	−0.4 (4)
C4—C5—C6—C7	−0.6 (5)	C7—C8—C9—N1	179.3 (3)
Cl1—C5—C6—C7	−179.5 (3)	O1—C8—C9—C4	179.6 (3)
C5—C6—C7—C8	−0.4 (6)	C7—C8—C9—C4	−0.7 (5)

### Hydrogen-bond geometry (Å, °)

**Table:**

*D*—H···*A*	*D*—H	H···*A*	*D*···*A*	*D*—H···*A*
O1—H1o···O2^i^	0.84 (1)	1.87 (1)	2.695 (3)	169 (4)
N1—H1n···O2	0.88 (1)	1.95 (1)	2.816 (3)	167 (4)

Symmetry codes: (i) −*x*+1, −*y*+1, −*z*+1.
